# ^13^C Methacetin Breath Test for Assessment of Microsomal Liver Function: Methodology and Clinical Application

**DOI:** 10.1155/2017/7397840

**Published:** 2017-07-05

**Authors:** Katarzyna Gorowska-Kowolik, Agata Chobot, Jaroslaw Kwiecien

**Affiliations:** ^1^Department of Pediatric Gastroenterology and Hepatology, Clinical Hospital No. 1, Zabrze, Poland; ^2^Department of Pediatrics, School of Medicine with the Division of Dentistry in Zabrze, Medical University of Silesia, Katowice, Poland

## Abstract

Assessment of the liver function, and the need of constant monitoring of the organ's capacity, concerns not only patients with primary liver diseases, but also those at risk of hepatopathies secondary to other chronic diseases. Most commonly, the diagnostics is based on measurements of static biochemical parameters, which allow us to draw conclusions only indirectly about the function and the degree of damage of the organ. On the other hand, liver biopsy is an invasive procedure and therefore it is associated with a considerable risk of complications. Dynamic tests enable us to assess quantitatively the organ's functional reserve by analyzing the kinetics of the metabolization of the substrate by the liver. In practice applied are breath tests using substances such as aminopyrine, caffeine, methacetin, erythromycin (for assessment of the microsomal function); phenylalanine, galactose (for assessment of the cytosolic function); methionine, octanoate, ketoisocaproic acid (for assessment of the mitochondrial function). The test with ^13^C methacetin belongs to the best described and most widely applied methods in noninvasive liver function assessment. Due to the rising availability of this method, knowledge concerning its limitations and controversies regarding the methodology, as well as its usefulness in chosen groups of patients, seems to be vital.

## 1. Introduction

The breath test using methacetin (methacetin breath test, MBT) belongs to dynamic tests that assess the microsomal function of the liver. They allow us to determine the enzymatic activity of the cytochrome P450 [1]. Its activity, which is responsible for the metabolism of many xenobiotics, decreases for example in chronic liver diseases, what is related to the negative effect of the proinflammatory cytokines [2]. Microsomal tests are considered to provide potentially the most information, that allow us to assess the so called functional mass of the organ. They are also characterized by the highest accuracy in assessing the long-term prognosis [1]. The use of carbon isotope ^13^C in the MBT (an isotope with no radioactive potential) increases the safety of the test and allows us to carry it out also in children and during pregnancy [3].

## 2. Assumptions and Methodology of the Test

The standard test is performed in a patient that remains fasting and the substrate is administered orally. After ingestion methacetin is absorbed from the gastrointestinal tract and is transported through the portal circulation to the liver, where it is broken into acetaminophen and ^13^CO_2_ by the enzymes of the cytochrome P450. The carbon dioxide containing the ^13^C isotope, that was created during the reactions, is eliminated with the exhaled air. Breath samples collected from the patient in adequate time intervals, are analyzed subsequently using mass spectrometry [3]. The results are compared to the basal (null) sample collected before ingestion of ^13^C methacetin. The increase of the content of ^13^C isotope in comparison to the total CO_2_ content in the exhaled air is estimated [4]. Noteworthy, the ^13^C isotope is naturally present in 1.11% of carbon atoms. Administration of the ^13^C labeled substrate can be therefore treated as simply increasing the physiologically present amount of the isotope in the body [5].

The increment in ^13^C concentration as ^13^CO_2_ content relative to the basal level is expressed as DOB (*delta over baseline*) and calculated according to the formula:

(1) $$\begin{array}{c} DOB=\delta \text{‰}{PDB}_{x}-\delta \text{‰}{PDB}_{0}, \end{array}$$

where *x* index indicates the sample collected after *x* minutes and 0 index pertains to the basal (null) fasted breath sample. δ‰PDB (*Pee Dee Belemnite*) is the unit determining the ^13^CO_2_ content within the total pool of the exhaled carbon dioxide relative to the international standard of the ^13^C:^12^C isotope proportion—the calcium carbonate of the fossil Belemnitella of the cretaceous Pee Dee formation is South Carolina (USA). Based on the obtained DOB results two basic parameters describing the kinetics of ^13^C methacetin metabolism are calculated:

1. cumulative dose of ^13^C methacetin recovered from the exhaled air (% *cumulative dose*, %CD), defined as the percentage of the exhaled ^13^CO_2_ relative to the amount of ^13^C methacetin that was administered. This value is considered to be the basic parameter for the assessment of the correctness of the MBT result,
2. time from administration of ^13^C methacetin to obtaining the peak elimination of ^13^CO_2_ in the exhaled air (*time to peak*, TTP), which reflects the kinetics of the process [6].

The mentioned parameters are presented as curves, reflecting the course of the reaction. Apart from the standard test, there is wide interest in the Breath ID method, which is based on a system of continuous breath analysis. The idea of Breath ID is to facilitate and increase the comfort of the test as well as raise the accuracy of the measurement at the same time. The samples are collected automatically every 3 minutes, through nasal canulas and under the control of a capnograph. Application of capnography allows us to avoid samples containing to low concentrations of CO_2_. The efficacy of this method is comparable with the standard test, what has been confirmed in a group of patients with various stages of liver cirrhosis in the course of hepatitis C [7].

Oral administration of methacetin results in high individual variability of the substrate's concentration, what is related to the absorption of methacetin from the gastrointestinal tract. This can further lead to lower accuracy of the obtained results. In order to avoid this limitation, the LiMAx (liver maximum function capacity) test was introduced. It is based on an automatic analysis of the air exhaled through a facial mask after intravenous administration of the substrate. The assumptions of this method are convergent with those of the standard test. Samples collected before as well as during 60 minutes after the administration of ^13^C methacetin are examined. The maximal function capacity of the liver is calculated according to the formula:

(2) $$\begin{array}{c} LiMAx=\frac{DOBmax\times R_{PDB}\times P\times M}{BW\;\left[\mu g/kg/h\right]}, \end{array}$$

where *R*_PDB_ represents the unit determining the ^13^CO_2_ content within the total pool of the exhaled carbon dioxide relative to the international standard of the ^13^C:^12^C isotope proportion—the calcium carbonate of the fossil Belemnitella of the cretaceous Pee Dee formation is South Carolina (USA); *P* is the initial value of CO_2_ production by the body, derived by multiplication of body surface area (m^2^) by the rate of 300 mmolCO_2_/h; *M* is mmol mass of ^13^C methacetin; BW is body weight of the examined individual.

Because of the increased precision of the results, this test is an effective—and in many places standard—diagnostic procedure in patients after liver surgery [8].

## 3. Factors Influencing the Test Results

The assumptions of the MBT cause that the activity of the examined enzymatic pathway is the main, but not the only factor influencing the final result. In order to calculate DOB a constant, baseline CO_2_ production by the body is presumed at the level of 300 mmolCO_2_/m^2^ body surface area/h. The increments of ^13^CO_2_ in the subsequently collected from the patient samples are compared to this value [5]. Therefore, the MBT result is influenced by all factors that increase the total amount of produced CO_2_ such as: age, physical activity, consumed meal or sparkling beverage, diseases of the respiratory tract, fever, and thyroid disorders [4, 9]. Zipprich et al. used MBT to study the influence of anemia and oxygen supplementation on the function of the cytochrome P450 in patients with liver cirrhosis. They based their hypothesis on the effect of hypoxia, which potentially impairs the hepatic metabolism, including the microsomal function of the liver. The study proved that in patients receiving oxygen (4 L/min), before and during the MBT a higher amount of ^13^C was recovered with the exhaled air than in patients breathing with atmospheric air. This effect was independent from the scores according to the Child–Pugh system and similar in patients with different stages of cirrhosis. There was also a correlation between the amount of the recovered ^13^C and the blood hemoglobin concentration. Based on the results the authors stated, that the hepatic clearance of methacetin depends not only on the function of the liver, but also on the degree of anemia and oxygenation, what is supposed to be related to their influence on the microsomal activity of the liver [10]. At the same time suggestions were risen, that the observed phenomenon may rather be related to the change of the total amount of exhaled CO_2_, than be caused by impact of oxygen supplementation on the activity of the cytochrome P450 [11].

An undisputable limitation of the MBT is the high hepatic extraction of methacetin, because of which methacetin metabolization depends on the perfusion of the liver by blood [1]. Studies concerning the association between the liver function capacity and the left ventricle ejection fraction (LV EF) as well as the dimensions of the left atrium in patients with chronic heart failure revealed that the CD of ^13^C methacetin after 120 minutes from the beginning of the test was related to the degree of heart failure assessed by NYHA staging system. The value of CD was significantly lower at all time points in patients with NYHA IV (who were characterized also by significantly lower LV EF) compared to patients with NYHA II and III as well as to the control group. The authors describe also a weak correlation between CD ^13^CO_2_ and LV EF as well as a weak invert relation between CD ^13^CO_2_ and the end-diastolic dimension of the LV. Moreover, the relationship between CD ^13^CO_2_ and the dimensions of the left atrium was statistically significant [12].

Another factor that may influence the results of MBT is a transjugular portosystemic shunt, which is often used to improve portal hypertension in end stage liver disease—a common complication of liver cirrhosis. It has been shown that the presence of a portosystemic shunt is able to reduce the metabolization rate of methacetin even if the liver function is normal [13].

In addition, in case of methacetin ingestion, the kinetics of the reaction was influenced to a great degree by the rate of the gastric emptying and the absorption of the substrate from the gastrointestinal tract [3, 4]. This phenomenon can be avoided by intravenous administration (as in the LiMAx test), what increases the precision of the obtained results [14].

It needs to be underlined too, that part of the ^13^C received by the patient takes part in metabolic processes or is built into tissues and is not eliminated from the body with the exhaled air. For this reason, breath tests are considered to be semi-quantitative. The degree of nonrespiratory elimination of ^13^C from the body depends on the patient's individual metabolic properties. In one study 25 healthy volunteers received methacetin intravenously and had DOB increments and serum ^13^C methacetin concentrations measured. The results showed even 30% differences in the obtained DOB values depending on the degree of the extrahepatic processes taking part in the elimination of the substrate from the blood. Attempts were made to modify the methodology of the test in order to avoid the impact of this phenomenon. Holzhutter et al. suggested an assessment consisting of two stages (called the 2DOB test). Firstly, the patient received intravenously a dose of ^13^C labeled bicarbonate and subsequently a standard dose of ^13^C methacetin. As a result, two curves presenting DOB are obtained. The first one results from the administration of H^13^CO_3_ and reflects the kinetics of ^13^CO_2_ exchange between serum and various tissues. The second curve represents the metabolism of methacetin given to the individual. Based on the results and on the elaborated mathematical model the authors defined the so called hepatic detoxification index. Its accuracy in assessment of the hepatic CYP1A2 activity and predictive value are potentially higher than those of the standard MBT. An advantage of the presented method was also shortening the examination to 30 minutes, but the elaborated procedure required and additional measurement of serum ^13^C methacetin concentration, which stays in obvious contrast with the idea of a noninvasive breath test. Also, the comparable effectiveness of both methods, especially in terms of monitoring the degree of liver damage in individual patients, was underlined [14].

## 4. Influence of Xenobiotics on Cytochrome P450 Activity

Current attempts to determine the potential influence of diverse exogenous substances on MBT results are undertaken. A study in 12 healthy volunteers revealed longer TTP and decreased amount of ^13^C recovered with the exhaled air between the 24th and 75th minute of the test, when an individual smoked cigarette at the day of examination. On the other hand, habitual smoking probably stimulates the activity of the cytochrome CYP1A2 [15]. Induction of this subunit of the cytochrome can be caused also by smoking marijuana [16]. A negative impact on the obtained CD was described after ingestion of alcohol, however the differences were not statistically significant, what may indicate that the effect of alcohol on the liver's microsomal activity in healthy people is weak [17]. An advantage of the test seems to be the fact that its results are potentially independent from various medications such as: glucocorticosteroids, barbiturates, spironolactone, cimetidine, allopurinol, and chosen cytostatics [4]. Opposite observations concern selected contraceptives. A negative influence of ethinylestradiol (EE) on cytochrome P450 activity was described. It was defined as a lower maximal ^13^CO_2_ content recovered during medication with EE. In women on EE the recovered ^13^C methacetin contents were independent form the phase of the menstrual cycle during which the MBT was performed [18].

## 5. Application of MBT in Clinical Practice

MBT is one of the best described and willingly used out of the known breath tests. Methacetin, compared to other substrates, is distinguished by the speed of its hepatic metabolization and lack of toxicity. Additionally, the test is easy to perform and cheap [19]. These factors led to an increasing interest in introducing this method for diagnostic standards. Together with the request for the use of MBT in clinical practice, also the number of studies trying to assess the reproducibility of its results as well as its diagnostic value in different diseases increases. Kasicka-Jonerko et al. tried to estimate the impact of repeated MBT on the potential stimulation of the cytochrome CYP1A2. Based on their observations, the CD obtained during the first 60 minutes of the test can be considered to be the parameter characterized by best reproducibility. The authors described also that maximal recovered ^13^CO_2_ content and CD increase in cases when the MBT is repeated within 2-3 weeks, but the significance of this phenomenon was not validated in individuals with impaired liver function [20].

The biggest experience in applying MBT concerns the assessment of patients with liver cirrhosis, especially that resulting from HCV infection. Several studies confirmed the method's usefulness in monitoring and assessment of the degree of liver cirrhosis in patients with hepatitis C [21–24]. According to Lazar et al. the MBT using BreathID is a good tool for assessment of the degree of inflammation and fibrosis also in those patients with hepatitis C, whose alanine aminotransferase activity remains within normal ranges [22]. As presented by most reports, MBT is also a better tool for the assessment of fibrosis and cirrhosis in patients with hepatitis C than the typically applied standard biochemical parameters [23]. MBT results allowed additionally to distinguish patients with established cirrhosis within the group of patients with primary biliary cirrhosis [24]. Moreover, studies concerning the usefulness of MBT in differentiating patients with nonalcoholic fatly liver diseases (NAFLD) and simple steatosis (SS) were carried out. They confirmed significant differences in the microsomal activity of the liver between patients with NAFLD and those with SS as well as the control group. The sensitivity and specificity of the test increase with the progression of fibrosis and are highest in stages F3-F4 [25]. Although latest studies have not described sufficient sensitivity of each of the MBT parameters in detecting fibrosis in patients with NAFLD, a high negative predictive value of the test for discriminating patients with NAFLD and fibrosis stage F1 or higher was confirmed. The authors suggest therefore that MBT should be included in the diagnostic algorithm of NAFLD as a tool allowing us to identify patients without fibrosis, who do not require liver biopsy [26]. MBT is also considered to enable the assessment of liver function in patients waiting for a liver transplant as well as monitoring the organ's function after the patient received the graft [27]. The test is additionally a promising alternative for pediatric patients. Until now MBT was positively tested among others in children with autoimmune hepatitis, obesity or NASH. It has been also proven, that in the pediatric age group MBT differentiates well chronic hepatitis without cirrhosis and those with established cirrhosis [6]. In the past years, the usefulness of MBT in distinguishing biliary atresia from other causes of infant cholestasis was shown too [28].

## 6. MBT in Selected Groups of Patients without Primary Hepatopathy

Individual studies concerned the application of MBT and interpretation of its results considering the specifics of some diseases. An attempt to analyze the impact of acute malnutrition in the course of anorexia nervosa (AN) on the hepatic metabolization of ^13^C methacetin revealed a different hepatic metabolism of the substrate in this group of patients. In individuals with AN significantly higher mean ^13^CO_2_ content recovered in the 120 minute of the test (%CD120) was obtained. The speed of the hepatic metabolism of ^13^C methacetin was significantly higher than in the control group as well as when compared to the recommended reference normal values for healthy people. The acceleration of the liver ^13^C methacetin metabolism (%CD120 > 37,3%) was present in more than half of the studied females with AN. These results may suggest an increased risk of false negative results in children with eating disorders and liver damage. This fact questions the reliability of the MBT in detecting potential liver function impairment in this group of patients [6].

A diverse metabolism of methacetin was described also within the elderly. In people aged above 65 years a significantly lower 120CD% than in younger people was shown (33.07% ± 7.06% versus 39.81% ± 5.68%). At the same time, TTP was comparable in both groups. The difference in 120CD% was even bigger after adjusting the presumed production of CO_2_ for age [9]. Opposite observations were presented for a middle-aged patient group (40–60 years old) compared to controls aged between 20 and 30 years. These findings stay in concordance with the described previously in the literature, age-related changes in the cytochrome CYP1A2 activity [29].

Furthermore, a study estimating the usefulness of MBT in patients treated with Extracorporeal Membrane Oxygenation (ECMO) was carried out. The LiMAx (maximum liver function capacity) test was performed, the substrate was administered intravenously, and the exhaled air samples were collected from the oxygenator. The breath test did not require changing the ECMO settings. The results correlated with the degree of liver capacity in the studied patients and the method was described as useful for the assessment of patients with multiorgan failure treated with ECMO [30].

## 7. Conclusions

Even though there are multiple markers of liver damage and liver capacity, still a method that would directly reflect the metabolic function of this organ and allow us to detect even slight changes of its capacity (being at the same time a minimally invasive and reproducible test) is lacking. The search for a noninvasive method that would enable optimal functional diagnostics of the liver is indisputably a challenge of the modern hepatology. In light of the presented research, MBT appears to be a useful tool for assessing the capacity of the organ, and as a prognostic marker in many diseases. The large individual variability concerning also the metabolism of cytochrome P450 can affect the results, but does not limit the usefulness of the method for monitoring patients. The impact of external factors on the activity of CYP1A2 subunit and their influence on the results of MBT are until now not fully understood and require further investigation.

## References

[1] Lalazar G., Ilan Y. (2009). Assessment of liver function in acute or chronic liver disease by the methacetin breath test: a tool for decision making in clinical hepatology. *Journal of Breath Research*.

[2] Nista E. C., Fini L., Armuzzi A. (2004). 13C-breath tests in the study of microsomal liver function. *European Review for Medical and Pharmacological Sciences*.

[3] Afolabi P., Wright M., Wootton S. A., Jackson A. A. (2013). Clinical utility of 13C-liver-function breath tests for assessment of hepatic function. *Digestive Diseases and Sciences*.

[4] Wieczorek S., Kempiński R., Poniewierka E. (2013). Application of breath testing in diagnostics of gastrointestinal tract [article in polish]. *Family Medicine & Primary Care Review*.

[5] Braden B., Lembcke B., Kuker W., Caspary W. F. (2007). 13 C-breath tests: current state of the art and future directions. *Digestive and Liver Disease*.

[6] Kwiecien J., Oswiecimska J., Bak-Drabik K., Gorowska-Kowolik K., Ziora K. (2014). 13C-methacetin breath test in the assessment of liver function in girls with anorexia nervosa [article in polish]. *Standardy Medyczne Pediatria*.

[7] Goetze O., Selzner N., Fruehauf H., Fried M., Gerlach T., Mullhaupt B. (2007). 13C-methacetin breath test as a quantitative liver function test in patients with chronic hepatitis C infection: continuous automatic molecular correlation spectroscopy compared to isotopic ratio mass spectrometry. *Alimentary Pharmacology & Therapeutics*.

[8] Stockmann M., Lock J. F., Riecke B. (2009). Prediction of postoperative outcome after hepatectomy with a new bedside test for maximal liver function capacity. *Annals of Surgery*.

[9] Ciccocioppo R., Candelli M., Di Francesco D. (2003). Study of liver function in healthy elderly subjects using the 13C-methacetin breath test. *Alimentary Pharmacology & Therapeutics*.

[10] Zipprich A., Meiss F., Steudel N., Sziegoleit U., Fleig W. E., Kleber G. (2003). 13C-Methacetin metabolism in patients with cirrhosis: relation to disease severity, haemoglobin content and oxygen supply. *Alimentary Pharmacology & Therapeutics*.

[11] Candelli M., Cazzato I. A., Nista E. C., Pignataro G., Gasbarrini A. (2003). 13C-methacetin breath test and oxygen supply. *Alimentary Pharmacology & Therapeutics*.

[12] Málek F., Hendrichová M., Krátká K., Sedlaková M., Vránová J., Horák J. (2008). Correlation of the functional liver mass with left ventricular ejection fraction and left atrial diameter in patients with congestive heart failure. *International Journal of Cardiology*.

[13] Candelli M., Pompili M., Suppressa P. (2012). Liver involvement in hereditary hemorrhagic telangiectasia: can breath test unmask impaired hepatic first-pass effect?. *Internal and Emergency Medicine*.

[14] Holzhütter H. G., Lock J. F., Taheri P., Bulik S., Goede A., Stockmann M. (2013). Assessment of hepatic detoxification activity: proposal of an improved variant of the 13 C-Methacetin breath test. *PloS One*.

[15] Kasicka-Jonderko A., Loska D., Jonderko K., Kaminska M., Błonska-Fajfrowska B. (2011). Interference of acute cigarette smoking with [13C] methacetin breath test. *Isotopes in Environmental and Health Studies*.

[16] Anderson G. D., Chan L. N. (2016). Pharmacokinetic drug interactions with tobacco, cannabinoids and smoking cessation products. *Clinical Pharmacokinetics*.

[17] Wutzke K. D., Forberger A., Wigger M. (2008). Effect of alcohol consumption on the liver detoxication capacity as measured by [^13^C]methacetin- and [methyl-^13^C]methionine-breath tests. *Isotopes in Environmental and Health Studies*.

[18] Jonderko K., Skalba P., Kasicka-Jonderko A., Kaminska M., Bizior-Frymus D., Dyja R. (2013). Impact of combined oral contraceptives containing ethinylestradiol on the liver microsomal metabolism. *The European Journal of Contraception & Reproductive Health Care*.

[19] Stravitz R., Ilan Y. (2016). Potential use of metabolic breath tests to assess liver disease and prognosis: has the time arrived for routine use in the clinic?. *Liver International*.

[20] Kasicka-Jonderko A., Nita A., Jonderko K., Kamińska M., Błońska-Fajfrowska B. (2011). C-methacetin breath test reproducibility study reveals persistent CYP1A2 stimulation on repeat examinations. *World Journal of Gastroenterology*.

[21] Fierbinteanu-Braticevici C., Papacocea R., Tribus L., Cristian B. (2014). Role of 13C methacetin breath test for non invasive staging of liver fibrosis in patients with chronic hepatitis C. *The Indian Journal of Medical Research*.

[22] Lalazar G., Pappo O., Hershcovici T. (2008). A continuous 13C methacetin breath test for noninvasive assessment of intrahepatic inflammation and fibrosis in patients with chronic HCV infection and normal ALT. *Journal of Viral Hepatitis*.

[23] Dinesen L., Caspary W. F., Chapman R. W., Dietrich C. F., Sarrazin C., Braden B. (2008). 13 C-methacetin-breath test compared to also noninvasive biochemical blood tests in predicting hepatic fibrosis and cirrhosis in chronic hepatitis C. *Digestive and Liver Disease*.

[24] Kochel-Jankowska A., Hartleb M., Jonderko K., Kaminska M., Kasicka-Jonderko A. (2013). 13C–methacetin breath test correlates with clinical indices of liver disease severity in patients with primary biliary cirrhosis. *Journal of Physiology and Pharmacology*.

[25] Fierbinteanu-Braticevici C., Plesca D. A., Tribus L. (2013). Role of 13C-Methacetin breath test for the non-invasive evaluation of nonalcoholic fatty liver disease. *Journal of Gastrointestinal and Liver Disease*.

[26] Kempinski R., Neubauer K., Wieczorek S., Dudkowiak R., Jasinska M., Poniewierka E. (2015). 13C-Methacetin breath testing in patients with non-alcoholic fatty liver disease. *Advances in Clinical and Experimental Medicine*.

[27] Petrolati A., Festi D., De Berardinis G. (2003). 13C-methacetin breath test for monitoring hepatic function in cirrhotic patients before and after liver transplantation. *Alimentary Pharmacology & Therapeutics*.

[28] Shteyer E., Lalazar G., Hemed N. (2013). Continuous 13C-methacetin breath test differentiates biliary atresia from other causes of neonatal cholestasis. *Journal of Pediatric Gastroenterology and Nutrition*.

[29] Kasicka-Jonderko A., Jonderko K., Chabior E., Błonska-Fajfrowska B. (2008). Exact profiles of 13CO2 recovery in breath air after per oral administration of [13C] methacetin in two groups of different ages. *Isotopes in Environmental and Health Studies*.

[30] Bednarsch J., Menk M., Malinowski M., Weber-Carstens S., Pratschke J., Stockmann M. (2016). 13C breath tests are feasible in patients with extracorporeal membrane oxygenation devices. *Artificial Organs*.

